# Clinical findings and outcome in feline tetanus: a multicentric retrospective study of 27 cases and review of the literature

**DOI:** 10.3389/fvets.2024.1425917

**Published:** 2024-07-16

**Authors:** Alice Dussaux, Laurent Fuhrer, Martin B. Dorner, Koen Santifort, An Vanhaesebrouck, Marika Menchetti, Cecilia-Gabriella Danciu, Guillaume Dutil, Catherine Escriou, Stephane Blot, Sarah Gutmann, Carina Taestensen, Vincent Mayousse

**Affiliations:** ^1^C.H.V. des Cordeliers, IVC Evidensia France, Meaux, France; ^2^Clinique Vétérinaire Saint Avertin, Fovéa, Saint-Avertin, France; ^3^Biological Toxins, Centre for Biological Threats and Special Pathogens, Robert Koch Institute, Berlin, Germany; ^4^Department of Neurology and Neurosurgery, Evidensia Animal Hospital Arnhem, Arnhem, Netherlands; ^5^Department of Veterinary Medicine, University of Cambridge, Cambridge, United Kingdom; ^6^Department of Neurology and Neurosurgery, Private Veterinary Clinic San Marco Srl, Veggiano, Italy; ^7^Department of Veterinary Clinical Science and Services, Royal Veterinary College, London, United Kingdom; ^8^Department of Veterinary Clinical Sciences, University of Agricultural Sciences and Veterinary Medicine of Cluj-Napoca, Cluj-Napoca, Romania; ^9^Division of Clinical Neurology, Department of Clinical Veterinary Medicine, Vetsuisse Faculty, University of Bern, Bern, Switzerland; ^10^Small Animal Internal Medicine Department, Neurology Service, VetAgro Sup, Lyon, France; ^11^Department of Neurology, Ecole Nationale Vétérinaire d’Alfort, CHUVA, Maisons-Alfort, France; ^12^Department for Small Animals, Leipzig University, Leipzig, Germany

**Keywords:** tetanus, cats, feline, *Clostridium tetani*, neurotoxin, muscle stiffness, tetanospasmin

## Abstract

Tetanus is a toxigenic illness caused by the action of *Clostridium tetani* neurotoxin (TeNT), which results in partial or generalized muscle stiffness in infected mammals and birds. The disease is rarely reported in cats due to their innate resistance to the toxin. This multicentric retrospective study aimed to describe a significant population of cats with a diagnosis of tetanus and report their signalment, clinical and neurological signs, diagnostic findings, treatment, and outcome. A retrospective search through medical records from 11 referral centers in Europe resulted in the identification of 27 cases of feline tetanus from July 2005 to April 2023. These cases were further compared with previously reported cases in the veterinary literature. Young cats were more commonly represented than older cats, with a median age of 4 years. Clinical signs were initially characterized by a lame and/or stiff limb, near the primary injury site, in 17/26 (65%) cats. Signs were focal or multifocal in 21/27 (78%) cats of this study and one typical sign was the inability to flex the most severely affected limbs. Electrodiagnostic studies revealed characteristic changes, such as continuous spontaneous motor unit discharges in both agonist and antagonist muscles. Such studies are particularly useful in focal and multifocal cases and should be performed to further support the diagnosis. The toxin was successfully identified in one case using the mouse bioassay. Treatment included antibiotherapy (metronidazole) in most cases, muscle relaxants, appropriate nursing cares and handling of potential complications. Overall, the outcome appeared to be positive, with only 1/27 (3.7%) cats being euthanized due to financial restrains. 23/25 (92%) cats returned to an independent ambulatory capacity on all limbs within a median delay of 25 days. Mild to moderate long-term sequelae were reported in eight (30%) cats. This multicentric study is the first to bring together such a large number of cats affected with tetanus. Presentation of the disease in cats differs from that observed in humans and dogs, with most cats being locally affected. Compared to previous reports of tetanus, this series of cats had a better outcome overall, especially for cats affected with generalized tetanus.

## Introduction

Tetanus is an acute toxigenic illness affecting mammals and birds caused by the action of a tetanic neurotoxin (TeNT; aka tetanospasmin) formed in the body during the vegetative growth of *Clostridium tetani*. Following penetration of the bacterial spores into a wound and after sporulation, tetanospasmin enters the axons of the adjacent motor nerves at the neuromuscular endplate, migrates by retrograde transport to the neuronal cell body within the spinal cord. The toxin may also ascend the spinal cord until it reaches the brain (1). An alternative route to the brain is via cranial nerves or direct penetration. It is then internalized by inhibitory interneurons in the spinal cord (Renshaw cells) and brainstem, where it will irreversibly inhibit the release of the inhibitory neurotransmitters glycine and gamma-aminobutyric acid. It results in generalized or partial muscle stiffness and spasms (1–3).

Cats are considered to be, respectively, 12 and 2,400 times more resistant to the toxin than dogs and humans (4). This phenomenon tends to predispose cats to more localized forms of the disease and explains a lower prevalence in comparison with other species (1, 5). To the authors’ knowledge there are no clinical studies describing tetanus in a substantial number of cats apart from several isolated case reports (6–24), short case-series (25, 26) or studies with experimentally infected cats (27, 28).

Thus, the aim of this study was to report a significant number of feline tetanus cases, with their signalment, clinical and neurological signs, diagnostic findings, treatment, and outcome and to compare them with previously described reports.

## Materials and methods

The medical records of cats diagnosed with tetanus at 11 veterinary referral institutions in Europe between July 2005 and April 2023 were reviewed. Cases were recruited via an online veterinary forum, asking for neurologists to participate in the study. As no validated definitive antemortem test exists for the disease in small animals, cats were included if they had characteristic clinical signs evaluated by a neurology specialist or by a resident under the supervision of a neurology specialist, without evidence of other neurological disease or lameness/stiffness due to orthopedic diseases. Information retrieved from the medical records included signalment, housing environment of the cat, clinical signs at the onset of the disease and their duration, presence of a potential source of infection, and findings from neurological examination.

Grading systems designed to facilitate the evaluation of the severity of tetanus are reported and commonly used in dogs (29) and humans (30). These systems share a lot of similarities considering that the progression and extent of the disease are quite alike in dogs and humans. Due to a different clinical presentation in dogs and humans compared to cats from our study, a more appropriate grading system was required.

Each case was subsequently classified into three categories depending on the extent and severity of tetanus. The first category was entitled “focal tetanus” and included all cases in which tetanus was localized to one muscle group or one part of the body (e.g., one limb, head only, tail only). The second category was “multifocal tetanus” and included cats with involvement of several parts of the body with no generalized involvement of the muscles. For example, a cat whose all 4 limbs would be affected with no extension to the facial muscles would be included in this category. A cat presenting with pelvic limbs stiffness and involvement of the facial muscles but with no involvement of the thoracic limbs would also be included in this category. The last category was “generalized tetanus,” which was further graded as “grade I” and “grade II.” Grade I generalized tetanus included cats with mild to moderate generalized muscular involvement in which feeding and micturition abilities were preserved. Grade II generalized tetanus included cats with severe generalized muscular involvement, preventing any feeding or micturition.

In human medicine, the term “local tetanus” is preferred to describe clinical signs of tetanus that are restricted to a limited area of the body (31). Considering the necessity to introduce an intermediate category named “multifocal tetanus” in the cats of this study, the term “focal” will be used in this text to qualify clinical signs that are localized to a single part of the body.

Results of diagnostic testing, including bloodwork, urine analysis, diagnostic imaging, electrodiagnostic study, cerebrospinal fluid (CSF) analysis, bacterial culture on wound material and serology, were recorded when available. Treatment protocols and nursing care provided during hospitalization or at home were collected as well as any kind of complications. For cases to be included, a minimum follow-up until return to an autonomous ambulatory capacity or, where available, until death or euthanasia was required. Development of medium to long-term sequelae, if any, was recorded and described.

## Results

### Signalment, clinical signs and epidemiological features

Twenty-seven cats with a diagnosis of tetanus were identified. One case had already been described in a case report (9). The most common breed in this cohort was domestic shorthair (*n* = 25). There were also one Abyssinian and one Bengal. There were two intact female cats, seven neutered female cats, four intact male cats and 14 neutered male cats. Age and weight were available for 24 and 26 cats respectively; median age was 4 years (range 0.6–12.1 years) and median weight was 4.25 kg (range 2.6–7.7 kg). Regarding seasonality, seven cases developed during spring (March–April–May), six cases during summer (June–July–August), seven cases during fall (September–October–November) and two cases during winter (December–January–February). The season was not specified in the remaining five cases. Most cats had outdoor access (21/27 cats). Three individuals were indoor-only cats. For the three remaining cats, this information was not available. Among the 21 outdoor cats, 9/21 lived in the countryside, 5/21 lived in a suburban area and 6/21 lived in the city.

The median duration from onset of clinical signs (as assessed by the owner) to presentation to a neurology service was available for 26 out of 27 cats and was 4 days (range 0–14 days).

Twenty-three cats were presented with an infected wound (*n* = 17) or history of a previous injury (*n* = 6). Most injuries were of non-iatrogenic origin: broken claw (2/23), skin erosions/wounds of unknown origin (10/23), open fractures (3/23), bites (2/23), and parturition (1/23). An iatrogenic cause was found in five cats (recent history of sterilization in four males and one female). Regarding the indoor-strict cats, 2/3 cats had a history of previous neutering and 1/3 cat had a broken infected claw.

Lameness and/or stiffness of one limb was the first clinical sign observed by the owners in 17/26 cats (Supplementary Video S1). Among these cases, most presented with a wound located on (*n* = 11) or near (*n* = 5) the lame/stiff limb. Further details regarding the initial clinical presentation and localization of the wound in each case are provided in Table 1.

**Table 1:** Feline tetanus severity classification and evolution of the clinical signs with time in relation to the localization of the wound (L = left, R = right, PL = pelvic limb, TL = thoracic limb, Ls = limbs).

Form of tetanus	Case	Localization of the wound (if any)	Localization of initial clinical signs	Localization of final clinical signs (when considering the worst extent of the disease)
Focal	1	LPL	LPL	LPL
	2	LPL	LPL	LPL
	3	RPL	RPL	RPL
	4	None found	General reluctance to move	RPL
Multifocal	5	RTL	RTL	Both TLs (L > R)
	6	Tail	RPL	4Ls (PLs > TLs)
	7	RPL	4Ls	4Ls (RPL > LPL > TLs)
	8	LPL	LPL	Both PLs (LPL > RPL), masticatory muscles
	9	Tail	RPL	4 Ls (PLs > TLs)
	10	Testis (castration)	LPL	4Ls (PLs > TLs)
	11	Abdomen	PLs	Both PLs
	12	Testis (castration)	LPL	Both PLs
	13	RPL	RPL	Both PLs (R > L), facial muscles
	14	Testis (castration)	PLs	Both PLs (L > R)
	15	None found	PLs	4Ls (PLs > TLs)
	16	Abdomen (spaying)	TLs	Both TLs
	17	None found	4Ls	4Ls
	18	None found	LTL	4Ls
	19	RTL	RTL	4Ls
	20	Abdomen	4Ls	4Ls
	21	LPL	LPL	4Ls (PLs > TLs), neck
Generalized (Grade I)	22	Testis (castration)	RPL	Mild to moderate generalized muscular involvement, able to feed and urinate independently
	23	Multifocal (more severe on the RTL)	RTL	Mild to moderate generalized muscular involvement, able to feed and urinate independently
	24	Reproductive tract (history of labor)	4Ls	Mild to moderate generalized muscular involvement, able to feed and urinate independently
	25	Multifocal (more severe on LPL)	Generalized	Mild to moderate generalized muscular involvement, able to feed and urinate independently
Generalized (Grade II)	26	LTL	LTL	Severe generalized muscular involvement, not able to feed and urinate independently
	27	LTL + back of the head and neck	LTL	Severe generalized muscular involvement, not able to feed and urinate independently, suspected partial and generalized seizures

In 18/27 cats, clinical signs progressively worsened, and 16/27 cats displayed evidence of spreading of the disease to other parts of the body. The median time elapsed between the onset of the first clinical signs observed by the owners and the time when the signs were most severe as assessed by a neurologist was available in 14 cats and was 4 days (range 1–7 days).

When considering the worst extent of the disease for each individual, clinical signs remained focal in four cats and were multifocal in 17 cats with involvement of either two (*n* = 7) or four limbs (*n* = 10). In 6/27 cats, signs spread to all muscles (Supplementary Videos S2, S3). A detailed description of the localization of the clinical signs for each case is available in Table 1.

For all cases, the severity of stiffness and increased muscle contraction was not homogeneous when considering each limb individually, and the most severe signs were always observed on the limb(s) in which clinical signs first developed (Supplementary Video S4).

The six cats which developed the generalized form of tetanus were considered to be grade I in four cases and grade II in two cases. In the latter, episodes of tachypnea and hyperthermia were reported. Focal and generalized tonic–clonic seizures with loss of consciousness were suspected in one of these two cats. The signs resolved following the administration of midazolam at admission and were not observed thereafter during hospitalization and the follow-up period.

The inability to flex the most severely affected limb(s) at least at one joint (usually the stifle joint or the elbow joint) was observed in all cats. This prevented testing of the spinal reflexes and postural reactions on these limbs in most cases, due to the excessive extensor tonus. In a few cats (*n* = 5) with involvement of the thoracic limbs, the carpal joint was maintained in a flexed position. Generalized or focal myalgia was reported in nine cats upon palpation of affected limb(s). Muscular fasciculations of the most severely affected limb(s) were also reported in three cats (Supplementary Video S5). Tail involvement was not detailed in most medical records. However, an erected tail was reported in three cats suffering from a generalized tetanus (two cats scored as grade I and one scored as grade II). In one case in which patellar reflex could still be elicited, an extreme hyperreflexia and a clonic response lasting 10–20 s were noticed (Supplementary Video S6).

Generalized anesthesia was performed in at least 19 cases. Among these, changes in tonus were only detailed in six cases, in which only a partial decrease in the abnormal muscular tone was observed. Finally, mild to marked hyperthermia [>39.2°C (32)] was reported in 10 cats.

### Diagnostic findings

Various diagnostic modalities were used in most cases to further support diagnosis of the disease and varied greatly between cases.

Bloodwork was performed in 24/27 cats and was unremarkable, except for a mild to severe increase in serum creatinine kinase (CK) activity in nine cats [range: 374–51,335 IU/L; reference interval (RI): 110–250 IU/L (33)]. Mild to marked increase in serum amyloid A (SAA) levels were observed in four cats [63.8–215 μg/mL; RI: <3.79 μg/mL (34)] as well as a mild to moderate neutrophilic leukocytosis in five cats (exact values not recorded). Further details on blood analysis findings are provided in Supplementary Table S1.

Urine analysis was performed in three cats and was unremarkable in all of them.

Several diagnostic imaging modalities [radiographs, ultrasounds, computed tomography and magnetic resonance imaging (MRI)] were used in several cats to rule out orthopedic, spinal and/or meningeal affections. Results from these tests are further described in Supplementary Table S1.

CSF analysis was performed in three cases and was unremarkable in two cases and highly contaminated with blood in one case.

Electrodiagnostic studies were performed in 12/27 cases. In three cases, the entire study was performed under general anesthesia. In two cases, electromyography was first performed on an awake cat and general anesthesia was then induced to further perform the nerve conduction studies. In these two cats, electromyography was briefly performed again under general anesthesia to evaluate if the anesthetized status of the animal could alter the study. In six cases, the cats were sedated with dexmedetomidine or medetomidine, then reversed with atipamezole. The electrodiagnostic study was performed while the cat was starting to wake up. In the remaining cat, no information was provided regarding the anesthetized status.

**Figure 1:**
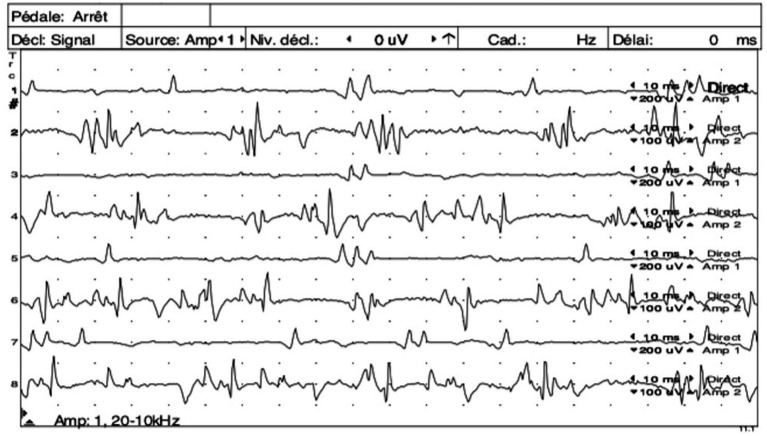
Simultaneous electromyography (EMG) tracings of the left quadriceps muscle (odd number tracings) and left semi-membranous muscle (even number tracings) showing continuous motor unit action potentials (MUAPs) as doublets, triplets and multiplets observed simultaneously in both antagonist muscles. Courtesy of École Nationale Vétérinaire d’Alfort.

In 11/12 cats, electrodiagnostic studies revealed continuous motor unit action potentials (MUAPs, Figure 1) that persisted after insertion of the needle, with 10 cats presenting simultaneous sustained activity in both extensor and flexor muscles. In one cat, whether the continuous MUAPs were observed simultaneously in both antagonistic and agonistic muscles was not specified. In most cats, these MUAPs consisted in a mix of single potentials, doublets, triplets and multiplets. In three cats, the MUAPs amplitudes were recorded and varied from 0.3 to 1.2 mV. Such amplitudes were higher when the animal was awake than when it was anesthetized, sometimes exceeding 1.5 mV. In the awake animals, presence of voluntary movements could sometimes interfere with the correct interpretation of the exam. In the same cats, the MUAPs frequency was recorded and varied from 120 to 160 Hz. The interval between two doublets was also calculated in these cats and varied from 1.5 to 2.5 ms. In cats affected with a focal or multifocal form, the MUAPs were only observed when testing the affecting limb(s) and disappeared when testing the normal limb(s). In 8/11 cats, these findings were only noticed when the animals were awake and were not observed anymore during general anesthesia or sedation.

Abnormal spontaneous electrical muscular activity (fibrillation potentials, positive sharp waves) was observed in one cat. The F wave/M wave amplitudes ratio was measured in one cat and was increased.

In one cat, electrodiagnostic was normal. In this cat, electrodiagnostic was performed under general anesthesia, after MRI.

Isolation of *Clostridium tetani* from anaerobic culture of the wound material was positive in all three cases tested. A definitive diagnosis was reached in one case, via detection of the TeNT in the patient’s serum by a mouse bioassay (MBA). The cat’s serum (450 μL) was injected intra-peritoneally into a female BALB/c mouse. The animal died within 15 h while a second animal receiving 450 μL of serum plus 50 μL of human anti-TeNT immunoglobulin (Tetagam P, 250 IU/mL, CSL Behring, Marburg, Germany) developed no signs. In the remaining 26 cases, a presumptive diagnosis of tetanus was established based on a combination of clinical presentation, presence or history of a wound/previous injury and results from additional ancillary tests.

### Treatments

21/27 cats were hospitalized following the diagnosis of tetanus. 4/27 cats were not hospitalized mainly due to financial restraints and no information could be found about a possible hospitalization in the remaining two cats. Median hospitalization time was 7 days (range: 1–59 days).

Wound disinfection and/or debridement were explicitly mentioned for 9/27 cats.

Antibiotics were administered in 26/27 cats, either as a monotherapy (*n* = 14), as a bitherapy (*n* = 11) or as a tritherapy (*n* = 1). Metronidazole was the most common type of antibiotics used in this population (*n* = 20; dosage range: 8–15 mg/kg/12 h), followed by the combination of amoxicillin and clavulanic acid (*n* = 7; dosage range: 12.5–20 mg/kg/12 h). Durations of antibiotic treatments were only available for seven cats receiving metronidazole and two cats receiving clindamycin. Among these cats, the median metronidazole treatment duration was 15 days (range 7–28 days). In one cat, clindamycin was administered for 6 weeks and in another cat, clindamycin was discontinued after 2 days once toxoplasmosis was ruled out. Further details about antibiotic treatments are provided in Supplementary Table S2.

Different combinations of various types of myorelaxants were used in most cats (*n* = 21), either as a monotherapy (*n* = 11), bitherapies (*n* = 7), or tritherapies (*n* = 2). Diazepam was the most common type of myorelaxants used in this population. To achieve myorelaxation, a combination of diazepam, methocarbamol, alfuzosin and magnesium sulfate was used in one cat. This cat suffered from marked urine retention and also received bethanechol. Other combinations of myorelaxants are further detailed in Supplementary Table S3.

Equine tetanus antitoxin was administered in 6/27 cats, three being affected with a multifocal form, two with a grade I generalized form and one with a grade II generalized form. Exact doses and administration routes were only available for three of these six cats [1 dose of 750 IU subcutaneously (SC) for the first cat, 1 dose of 300 IU/kg SC then 1 dose of 700 IU/kg intravenously (IV) in the second cat and 2 doses of 100 IU/kg IV 48 h apart for the third cat]. No side effects were reported in any of the cats following antitoxin administration.

Additional treatments are further detailed in Supplementary Table S3.

### Complications

Complications were observed in 14/27 cats, including mild to severe consequences depending on the cases. Most complications were reported in multifocal and generalized forms. Among these cases, the most common complications included episodes of hyperthermia (*n* = 10), mild to marked urine retention (*n* = 4), osteoarticular diseases (*n* = 4) and dysphagia (*n* = 3). Regarding focal cases, transient constipation was observed in one cat and was the only reported complication. Further adverse events are detailed in Table 2.

**Table 2:** Complications and adverse events observed secondary to tetanus or to provided cares.

Complications (*n* = 14/27)	Number of cats affected	Tetanus grade	Specific treatments and cares provided
Mild urine retention	2	Multifocal (*n* = 2)	Manual bladder expression (*n* = 1) Various types of myorelaxants (*n* = 1)
Marked urine retention	2	Multifocal (*n* = 1) Grade II generalized (*n* = 1)	Various types of myorelaxants (*n* = 2) Urinary catheterization (*n* = 2) Sphincterotomy (*n* = 1, after 3 weeks of urine retention)
Constipation	2	Focal (*n* = 1) Multifocal (*n* = 1)	None (*n* = 1) Oral lactulose and rectal enema (*n* = 1)
Diarrhea	1	Multifocal	Smectite
Dysphagia	3	Grade II generalized (*n* = 2) Multifocal (*n* = 1)	Metoclopramide (*n* = 1) Nasogastric feeding tube (*n* = 2) Gastrotomy feeding tube (*n* = 1)
Intermittent tachypnea	1	Grade II generalized	None
Bradycardia	1	Grade II generalized	None
Hyperthermia (up to 41°C)	10	Multifocal (*n* = 5) Grade I generalized (*n* = 3) Grade II generalized (*n* = 2)	None (apart from antibiotherapy and use of myorelaxants and analgesics)
Generalized phlebitis secondary to repeated catheterization and prolonged hospitalization (oral treatments were not possible due to the cat’s marked aggressivity)	1	Multifocal	Application of chlorhexidine-soaked bandages
Patellar luxation	1	Multifocal	Trochleoplasty
Coxofemoral luxation	1	Multifocal	Conservative treatment (financial constraints)
Radial and ulnar osteomyelitis at the level of the wound, leading to pathological fracture (*n* = 1) and tarsal luxation (*n* = 1)	2	Grade I generalized (*n* = 1) Grade II generalized (*n* = 1)	Antibiotics and wounds debridement, initially (*n* = 2) Amputation of the limb (*n* = 2)
Corneal ulceration	1	Multifocal	Not specified
Mild pleural effusion suspected to be secondary to central veinous catheterization	1	Multifocal	Not specified

### Outcome

Follow-up duration was available for 25/27 cats. The exact follow-up period was not specified for the two remaining cats. The follow-up time greatly varied between cases, with a median of 47 days (range: 1–5,110 days). One cat was euthanized 1 day following admission, due to financial restraints. Excluding the latter and one cat which always remained ambulatory, 23/25 (92%) cats recovered an independent ambulatory capacity on all limbs within a median period of 25 days (range: 11–45 days). Amputation of a limb secondary to the primary injury (osteomyelitis) was the reason for the failure to recover an acceptable ambulatory capacity on all limbs in the two remaining cats. These cats recovered an ambulatory capacity on three limbs in 21 and 83 days, respectively. Details regarding time to recover an independent ambulatory capacity in relation to the tetanus severity are presented in Table 3.

**Table 3:** Time to return to an independent ambulatory capacity and possible reported sequelae in relation to the tetanus form presented by the cats of this study, excluding one cat that was euthanized upon admission due to financial restraints (Ls = limbs, LPL = left pelvic limb, PLs = pelvic limbs).

Case n°	Tetanus form	Time to return to an independent ambulatory capacity (days)	Sequela(e) (from tetanus or from primary injury)
1	Focal	Within 21	Not able to jump as high as before
2	Focal	Within 25	None
3	Focal	21	None
4	Focal	15	None
5	Multifocal (2Ls)	Within 22	None
6	Multifocal (2Ls) + masticatory muscles	Within 28	None
7	Multifocal (2Ls)	Within 21	None
8	Multifocal (2 Ls)	Within 19	None
9	Multifocal (2 Ls + facial muscles)	16	Persistent paraparesis
10	Multifocal (2 Ls)	34	Marked LPL lameness (coxofemoral luxation)
11	Multifocal (2 Ls)	21	None
12	Multifocal (4 Ls)	45	PLs muscles atrophy
13	Multifocal (4 Ls)	25	None
14	Multifocal (4 Ls)	31	Right stifle arthrosis (patellar luxation)
15	Multifocal (4 Ls)	30	None
16	Multifocal (4 Ls)	37	None
17	Multifocal (4 Ls)	37	None
18	Multifocal (4 Ls)	28	None
19	Multifocal (4 Ls)	28	None
20	Multifocal (4 Ls + neck)	Within 45	None
21	Generalized (grade I)	Within 45	Possible REM-sleep disorder (not observed after 4 months since first presentation) Persistent RPL stiffness
22	Generalized (grade I)	11	None
23	Generalized (grade I)	83	LPL amputation
24	Generalized (grade I)	All time ambulatory	None
25	Generalized (grade II)	21	LTL amputation
26	Generalized (grade II)	40	None

Medium to long-term sequelae directly related to tetanus were reported in 6/26 cats. These included pelvic limbs muscle atrophy (*n* = 1), a decreased jumping ability (*n* = 1), muscle fibrosis and decrease in the articular range of motion of one limb (*n* = 1), stifle arthrosis (*n* = 1), persistent ambulatory paraparesis (*n* = 1), and marked pelvic limb lameness (*n* = 1). Further details are available in Table 3. One cat also developed transient episodes of muscular hyperactivity during sleep, which were suspected to be associated with a rapid-eye-movement (REM) sleep disorder (Supplementary Video S7). These episodes were always observed during sleep, were not associated with autonomic signs, and could be easily interrupted by waking up the cat. Upon last recheck 4 months following first presentation, these abnormal episodes were no longer observed by the owners.

## Discussion

This retrospective study includes the largest reported population of cats clinically diagnosed with tetanus, describing the signalment, clinical, neurological and diagnostic findings, treatment and outcome. Tetanus in cats has already been reported previously in the veterinary literature. All previously reported cases of cats naturally affected with the disease (6, 7, 10–24) will be referred to as the “historical cases” in this paper to differentiate them from the clinical cases described above.

Among clinical and historical cases, the most common breed was the DSH cat. Neutered male cats represented 51. 8% of total individuals in this study but only represented 11. 5% of affected cats in previous reports (Supplementary Table S4). This population mainly consisted of young cats with a median age of 4 years, similar to what has been described in previous studies in dogs (35). This is also in accordance with the historical cases, for which the median age at the time of infection was 2 years. Most cats had outdoor access, with a majority of individuals living in the countryside or in a suburban area. It is worth noticing that three cats were indoor-strict cats and still developed tetanus. In these cats, iatrogenic contamination (2/3 cats had a recent history of neutering) or contamination of the non-iatrogenic or surgical wound from household environment were suspected sources of infection.

In dogs the prevalence of tetanus appears significantly higher during winter months according to one English study (36). Only 2/22 cats from our study in which the information was available presented clinical signs of tetanus during winter. The remaining cases were equally distributed within the three other seasons. This difference might be due to regional variations or more probably to inherent differences existing between feline and canine habits regarding outdoor access frequency. Indeed, one study demonstrated that the average hourly physical activity in cats was greater when days were long (summer days) than when days were short (winter days) (37), which can result in a reduction of the time spent outside during the winter season and thus in a reduced exposition to potential injuries. Such a variation might be less pronounced in dogs, as their outdoor access frequency often depends on the owner, which seems to remain constant throughout the year, as demonstrated by Lail et al. (38).

A wound, a recent injury or a surgical intervention was reported in 23/27 (85%) cats in this study and in 25/26 (96%) cats from historical cases (Supplementary Table S5). In all cats, clinical signs were first observed near the injured area. They were initially characterized by a lame and/or stiff limb in 17/26 (65%) cats of this study and in 14/26 cats of historical cases (54%). This differs from dogs in which the most common initial signs were ocular and facial signs (29) and from humans, who usually display neck stiffness, sore throat and difficulty opening the mouth as early symptoms (31). In our cases, if the progression of the disease occurred, the muscular stiffness was always more severe and long-lasting in the area where clinical signs were first observed. This could be explained by the higher concentration and thus binding and entry of the TeNT in motor neuron terminals around the site of toxin release (39).

Progression and extent of the disease are quite alike in dogs and humans (29, 30), and quite different from the clinical presentation observed in cats from this study. Indeed, in contrast with dogs (35, 40) and humans (31) in which the generalized form is the most common form of tetanus, it was only reported in six (22%) cats from our study. The proportion of generalized presentations was higher in historical cases, with 11/26 (42%) cats being affected with the generalized form. Involvement of facial muscles were only observed in eight (30%) cats in this study and in 13 (50%) cats from previous case reports. These discrepancies are likely due to the inherent higher resistance of cats to the tetanus toxin compared to dogs and humans. Among clinical and historical cases classified in the “multifocal tetanus” category, 10/25 cats, (40%) presented with their four limbs affected, with no involvement of facial/head muscles. Among cats which developed the generalized form, only two (33%) cats of this study were classified as grade II. One of these cats presented with a wound located at the base of the head and neck. In humans, this would predispose the individual to the rapid development of cephalic tetanus, which is associated with a high mortality (39). In historical cases, 6/11 (55%) cats were classified as grade II, while the other five cats were classified as grade I.

Generalized or partial myalgia was a common finding in cats of this study (9/27, 33%), which had been occasionally reported previously in 5/26 cats (19%). In human medicine, several hypotheses have been put forward to explain the origin of such pain. It could be the result of the intense and frequent muscle spasms observed in patients (31) and/or the consequence of a sensory peripheral neuropathy that is frequently observed in humans secondary to tetanus (41, 42). To the authors’ knowledge, this has not been explored in small animals.

In dogs, clinical signs of tetanus usually develop between 3 and 18 days after an injury (1, 35). Incubation times longer than a month are also not infrequently reported in humans and are related to the fact that spores may persist within the wound for several weeks before germinating when favorable conditions are met (39). Unfortunately, in this study as in previously reported cases, the duration between occurrence of injury and development of first clinical signs could not be precisely determined in most records. This was mainly because most cutaneous wounds went unnoticed by the owners and/or because numerous cats came back home wounded after several days spent outside.

In humans as in animals, diagnosis of tetanus is based on a history of a previous injury (and insufficient vaccination records in humans), along with typical clinical signs (2, 29, 31, 35, 40, 43, 44). Though the symptoms of the generalized form of tetanus are pathognomonic in humans as in animals, a significant focal increase in muscle tone along with episodic spasms affecting both agonist and antagonist muscle groups of one or several limbs should also raise suspicion of focal/multifocal tetanus. In all cats from this study and in most previously reported cases (10–13, 20, 21, 24, 25), this increase in muscle tone was characterized by an inability to flex the most severely affected limb(s) at least at one joint, preventing testing of the spinal reflexes and postural reactions on these limbs in most cases. Differential diagnoses of tetanus may vary depending on the form of the disease (focal, multifocal or generalized). They include myotonia or pseudomyotonia, polymyositis, strychnine poisoning, hypocalcemia, and central nervous system disorders with upper motor neuron signs mimicking tetanus (3). Such afflictions could be excluded in most cases by normal bloodwork and/or normal diagnostic imaging and/or results from electrodiagnostic studies and/or typical clinical history and resolution of the signs with treatment.

Bloodwork is usually normal or may reveal unspecific changes such as a neutrophilic leukocytosis [observed in five cats (18.5%) in this study] or an increase in CK [observed in nine cats (33%) in this study and in five cats (19%) from historical cases (Supplementary Table S6)] and aspartate aminotransferase (not evaluated in any cats) activity (2). Diagnostic imaging is also usually unremarkable in tetanus and was mainly used to exclude other orthopedical or neurological diseases in the cats of this study, especially because lameness was often the first sign observed.

Electrodiagnostic studies may help in diagnosing tetanus when displaying characteristic (although not pathognomonic) changes. Although their utility might be debatable in animals presenting with the typical clinical signs of a generalized form of tetanus, electrodiagnostic studies are particularly useful in focal or multifocal cases, especially at the onset of the disease, when the clinical signs are still moderate. In previous reports, typical findings included prolongation of needle insertion activity with subsequent continuous spontaneous activity and more importantly simultaneous motor unit discharges and doublets in both agonist and antagonist muscles. These changes are the results of the spontaneous firing of hyperactive motor neurons secondary to their defective glycinergic inhibition (12, 45). Similarly, these EMG changes were observed in 11/12 (92%) cats in this study. Interestingly, these findings persisted in two cats while being anesthetized but disappeared in eight cats during general anesthesia. Additionally, in one cat presenting with typical signs of tetanus, the electrodiagnostic studies were normal. This cat had been anesthetized for a long time before undergoing the exam. This is in agreement with the observation that some cats in this study (6/19, 32%) displayed a partial improvement of the clinical signs while under anesthesia. However, the lack of electrodiagnostic anomalies was not reported in previous case reports of feline tetanus describing electrodiagnostic studies in anesthetized cats (7, 10–12, 25). Pending further studies clarifying this controversial observation, general anesthesia should be avoided in cats with presumed tetanus that undergo electrodiagnostic studies.

In one cat, an increase in the F/M amplitudes ratio was recorded. This electrodiagnostic finding has been previously described in two cats suffering from a focal tetanus and suggests a disinhibition of the spinal motor neurons (7).

Culture and isolation of *Clostridium tetani* was performed in three cats of this study and in three cats from historical cases and was positive in all of them. In humans, culture of the bacteria is rarely performed considering the typical signs exhibited by the patients and because the entry route of the bacteria can be inapparent (46). Isolation of the bacteria is also often difficult (only 30–54% of success) (2, 46). Strictly speaking, the presence of *Clostridium tetani* in the wound does not prove the diagnosis—even so it is highly suspicious—as non-toxigenic strains of *Clostridium tetani* have been reported in the past (47, 48).

In one cat from our study, detection of the TeNT in the serum was attempted via the MBA and proved to be successful. This is the first time that such a test is reported on blood in feline medicine. This test had previously been reported on wound culture in two cats (19, 22). In humans, mouse bioassays have been used for over a century for the detection of clostridial neurotoxins (*botulinum and tetani*). This test is very sensitive and can detect as little as 6 pg./mL of TeNT (4). Still, diagnosis of tetanus is hampered by the frequently low levels of TeNT in blood circulation, related to the small infective dose required to induce clinical disease in people (2, 5, 47). As a considerably higher load of clostridial neurotoxin is required to induce the disease in cats, toxin detection tests may lead to higher rates of success in feline population compared to humans. However, further studies may be warranted to validate this procedure and to investigate the usefulness and welfare of such tests.

In human medicine, the treatment of the patient with tetanus aims to focus on three goals: prevention of further toxin absorption, elimination from the organism, and supportive care in an intensive care unit setting (44). Rareness of tetanus in small animals prevent practitioners from having official guidelines regarding treatment. In addition to great discrepancies regarding the severity of clinical signs between cases, treatment protocols were quite eclectic in our study. Despite this observation, treatment protocols described in our study aimed in most cases to complete these three objectives.

Equine tetanus antitoxin was administered in only six (22%) cats from this study, presenting with various degree of severity of clinical signs, and all of them survived. In people, human tetanus immune globulin is always given as soon as the disease is suspected to avoid further absorption of the toxin. This protocol has been reported to modestly reduce case fatality rates (44). In dogs, no difference in survival, severity or duration of clinical signs was observed in one study between dogs treated and not treated with antitoxin (35). As no patient died from tetanus in our study, whether receiving the antitoxin or not, the benefit of using such treatment in cats seems debatable, especially considering the potential risk of secondary anaphylactic reaction. Indeed, Godwin (17) described a cardiorespiratory arrest in a cat affected with generalized tetanus immediately following the intravenous administration of 5,000 units of equine tetanus antitoxin. Further studies are thus necessary to investigate the value of antitoxin treatment in cats, especially in individuals presenting with a severe generalized form of tetanus.

Complications were reported in 14 (51.9%) clinical cases, either directly related to tetanus or indirectly and secondary to the provided cares. They included mild to marked dysuria, constipation, diarrhea, tachypnea, bradycardia, osteoarticular issues, corneal ulceration, pleural effusion and generalized phlebitis.

Autonomic signs were very uncommon in this study compared with what has been reported in humans (2, 31, 42, 44) or in dogs (29, 35). Autonomic signs usually develop several days after the onset of the first spasms, usually persist for 1–2 weeks (31) and are associated with increased mortality rates in humans and dogs (29, 31, 44). These signs include hypertension, hyperthermia, tachycardia, arrhythmias, profuse sweating and less commonly bradycardia (35). The scarcity of such signs in cats from our study may be attributed to the low prevalence of severe generalized forms of tetanus, as autonomic signs are more commonly associated with higher severity grades of tetanus in humans (31) and dogs (29). Indeed, episodes of tachypnea and bradycardia were only reported in both cats that presented with a grade II generalized tetanus. Similarly, the occurrence of obvious autonomic signs was not previously reported in cats, except in one recent case report describing a cat with a severe generalized form of tetanus, which presented with ventricular arrythmias and died of a cardiopulmonary arrest (20). However, this finding should be interpreted with caution considering that the lack of evidence of autonomic signs might also be due to a possible lack of monitoring of such parameters. Furthermore, care should be taken to avoid confusing signs that might be related to morbidity-associated stress and pain with purely autonomic signs, especially in easily stressed species such as cats. Further prospective studies are therefore warranted to evaluate the true incidence of autonomic signs in feline tetanus.

Respiratory complications such as aspiration pneumonia and upper airway obstruction (due to pharyngeal and laryngeal spasms) are also common in human and canine tetanus and are a major cause of mortality in both species (31, 39, 43, 49). In this study, apart from intermittent episodes of polypnea reported in one cat, no major respiratory complications were reported in any cat, even for animals suffering from a grade II generalized form of tetanus. Among historical cases, respiratory difficulties followed by a respiratory arrest were reported in one cat affected with generalized tetanus (grade II) (25), which was successfully resuscitated.

Another difference with humans and dogs was the relatively common occurrence of osteoarticular issues in this study, either directly related to the chronically increased limbs muscular tone, or due to the primary infection which did not respond favorably to treatment. Fractures and luxations are rare complications of tetanus that have been sporadically reported in humans (50–54), and dogs (40, 55), as a consequence of prolonged muscle spasm. In one human study (54), the incidence of osteoarticular complications was 1. 8%, with most people presenting with thoracic vertebral fractures resulting from the prolonged and severe opisthotonos seen in generalized tetanus. In our study, one cat suffered from a right patellar luxation and the other presented with a left coxofemoral luxation. To the authors’ knowledge, such osteoarticular complications have never been described in cats secondary to tetanus. Both cats presented with a multifocal tetanus mainly affecting the pelvic limbs. It can be speculated that cats might be prone to osteoarticular complications affecting limbs compared to humans, given their predisposition to focal forms of tetanus.

In one cat affected with a generalized form of tetanus, partial and generalized tonic–clonic seizures were suspected. This cat had no history of previous epileptic episodes, and no other cause could be identified on bloodwork, CT scan and CSF analysis. Epileptic seizures have been reported previously in two cats affected with grade II-generalized tetanus (20, 25) as well as other animals and humans affected with tetanus (5, 29, 40, 56) and are suspected to be caused by a toxin-induced blockade of inhibitory synapses at a cortical level (57–59). In dogs, seizures may be observed in individuals graded as class III or IV on the tetanus severity classification system, which are the most severe grades (29).

Outcome was excellent for cats of this study, with only one (3.7%) cat being euthanized 1 day following admission and only motivated by financial restraints. The mortality rate was higher in historical cases, with eight (30.7%) cats being euthanized (4/8, 50%) or dying from a cardiopulmonary arrest (4/8, 50%) few days after their presentation to a veterinary clinic. Except for one cat which suffered from a focal form of tetanus and which was euthanized due to a lack of improvement of the signs after 3 days, all the remaining cats suffered from a grade I (1/7 cats) or II (6/7 cats) generalized form of tetanus. Except for two of these cats (6, 20), all the remaining cases were described in case reports published before the 90s. In comparison, all the clinical cases described in this study were cared for by board certified veterinary neurologists after 2005, which may have contributed to the difference in outcome between clinical and historical cases, due to the major advances made by the veterinary medicine over the last two decades. Alternatively, this difference could also be related to the low number of cases affected with generalized forms.

In this study, excluding the cat that had been euthanized upon admission, 24/26 (92%) cats regained an autonomous ambulatory status on all limbs within a median of 25 days. Amputation of a limb secondary to osteomyelitis at the site of the wound was the reason for not recovering a full ambulatory capacity in two cats. Median to long-term sequelae were reported in 6 (23%) cats and were mostly related to persistent gait deficits such as variable degree of lameness, persistent muscular rigidity and persistent mild paraparesis. Similar sequelae were described in 4/18 (22%) cats from historical cases. One cat from this study also developed transient episodes of muscular hyperactivity during sleep, suspected to be associated with a REM-sleep disorder. This is the first time that such a disorder is reported in a cat. The latter has been described in dogs as a common sequel to canine tetanus, being identified in at least 46% dogs in one recent study (60). In these dogs, the clinical signs spontaneously resolved within 6 months in 43% cases. In the cat from our study, these abnormal episodes were no longer observed within 4 months following discharge.

The limitations of this study include the inherent nature of a multicentric and retrospective study, which prevented any standardization of the diagnostic methods and treatment protocols. Gathered information might also have been incomplete. As tetanus is basically a clinical diagnosis and as no official diagnostic tests exist in the feline species, a definitive diagnosis was only obtained for one cat. Interestingly, even in humans such a test is rarely performed due to difficulties in obtaining conclusive results. As such, diagnosis of tetanus in most cats was presumptive, based on characteristic history and clinical signs (and their progression), exclusion of other causes and response to treatment.

## Conclusion

This retrospective study includes the largest reported population of cats diagnosed with naturally occurring tetanus, which was compared to previously reported cases in the veterinary literature. Young cats were more likely to be affected than older cats. Clinical signs were initially characterized by a lame and/or stiff limb in 65% cats, near the primary injury site, and remained focal or multifocal in 77% cats of this study. Diagnosis should be based on typical clinical signs such as the progressive impossibility to flex the most severely affected limbs due to excessive extensor tone, especially in the presence of a wound. In case of focal/multifocal forms of tetanus, electrodiagnostic studies should be performed to further corroborate the diagnosis. Overall, the outcome appeared to be positive, with only one cat being euthanized due to financial restrains and with most cats returning to an independent ambulatory capacity on all limbs. Prognosis should be guarded for grade II-generalized forms of tetanus, as most cats affected with this form died from cardiopulmonary arrest or were euthanized in previously reported cases. Mild to severe long-term sequelae were reported in 23% cats.

## Data availability statement

The original contributions presented in the study are included in the article/Supplementary material, further inquiries can be directed to the corresponding author.

## Ethics statement

Ethical approval was not required for the studies involving animals in accordance with the local legislation and institutional requirements because to our institution, there is no need to obtain an ethical approval for retrospective studies. Written informed consent was not obtained from the owners for the participation of their animals in this study because the cases were retrospectively reviewed. Written informed consent was obtained from the individual(s) for the publication of any potentially identifiable images or data included in this article.

## Author contributions

AD: Conceptualization, Investigation, Methodology, Resources, Writing – original draft, Writing – review & editing, Visualization. LF: Resources, Supervision, Validation, Writing – review & editing. MD: Investigation, Supervision, Validation, Writing – review & editing. KS: Resources, Supervision, Validation, Writing – review & editing. AV: Resources, Supervision, Validation, Writing – review & editing. MM: Resources, Writing – review & editing, Supervision, Validation. C-GD: Resources, Supervision, Validation, Writing – review & editing. GD: Resources, Supervision, Validation, Writing – review & editing. CE: Resources, Supervision, Writing – review & editing. SB: Resources, Writing – review & editing. SG: Resources, Writing – review & editing, Validation. CT: Resources, Writing – review & editing, Validation. VM: Funding acquisition, Methodology, Resources, Supervision, Validation, Visualization, Writing – review & editing.

## Supplementary material

SUPPLEMENTARY VIDEO S1 Extreme right pelvic limb stiffness in a cat affected with focal tetanus.

SUPPLEMENTARY VIDEO S2 Generalized stiffness in a cat affected with grade I generalized tetanus. Flexion of the limbs is not possible, except for the carpal joints that are maintained in a flexed position.

SUPPLEMENTARY VIDEO S3 Generalized stiffness in a cat affected with grade II generalized tetanus. Flexion of the limbs is not possible, except for the carpal joints that are maintained in a flexed position.

SUPPLEMENTARY VIDEO S4 Neurological exam at admission of a cat affected with grade I generalized tetanus. Severity of stiffness is more pronounced on the pelvic limbs, where signs were first observed, than on the tail, thoracic limbs and facial muscles. Flexion of the pelvic limbs is not possible. Courtesy of Koen M. Santifort.

SUPPLEMENTARY VIDEO S5 Pelvic limbs muscular fasciculations observed in a cat affected with 4 limbs multifocal tetanus. Signs were more severe on the pelvic limbs.

SUPPLEMENTARY VIDEO S6 Hyperreflexia and clonic response elicited after patellar reflex testing in a cat affected with grade I generalized tetanus. Courtesy of Koen M. Santifort.

SUPPLEMENTARY VIDEO S7 Sleep-associated episode of muscular hyperactivity suspected to be associated with a rapid-eye-movement sleep disorder observed in a cat upon recovery from a grade I generalized tetanus. Courtesy of Koen M. Santifort.
