# Supplementary material for: Clinical findings and outcome in feline tetanus: a multicentric retrospective study of 27 cases and review of the literature
Source: Front Vet Sci. 2024 Jul 16;11:1425917. doi: 10.3389/fvets.2024.1425917 (PMC11286588; doi:10.3389/fvets.2024.1425917)
Supplement: Supplementary file 8 [file Table_1.DOCX]

Supplementary table 1. Results from complementary blood analysis and diagnostic imaging tests performed in the cats. MRI = magnetic resonance imaging, CT = computed tomography, FIV = feline immunodeficiency virus, FeLV = feline leukemia virus.

| **Blood analysis** |  |  | **n= 27 (100%)** |
| --- | --- | --- | --- |
|  | **Hematological findings** |  | |
|  |  | Unremarkable | 16 (59) |
|  |  | Not performed | 4 (15) |
|  |  | Neutrophilic leukocytosis | 5 (19) |
|  |  | Mild anemia | 3 (11) |
|  | **Biochemistry results** |  | |
|  |  | Unremarkable | 9 (33) |
|  |  | Not performed | 3 (11) |
|  |  | Increase in serum creatinine kinase activity | 9 (33) |
|  |  | Increase in serum amyloid A | 4 (15) |
|  |  | Increase in creatinine and urea | 1 (4) |
|  |  | Increase in urea | 2 (7) |
|  |  | Hyperlactatemia | 2 (7) |
|  |  | Hyperglobulinemia | 2 (7) |
|  |  | Increase in alanine aminotransferase activity | 2 (7) |
|  |  | Hypertriglyceridemia | 1 (4) |
|  |  | Hypoalbuminemia | 1 (4) |
|  | **FIV/FeLV status** |  | |
|  |  | Unremarkable | 2 (7) |
|  |  | Not performed | 25 (93) |
| **Diagnostic imaging** |  |  | |
|  | **X-rays findings (limbs)** |  |  |
|  |  | Unremarkable | 4 (15) |
|  |  | Not performed | 19 (70) |
|  |  | Soft tissue swelling | 1 (4) |
|  |  | Bone fracture | 3 (11) |
|  | **X-rays findings (thorax)** |  | |
|  |  | Unremarkable | 5 (19) |
|  |  | Not performed | 20 (74) |
|  |  | Cardiomegaly | 2 (7) |
|  |  | Diffuse bronchial pattern | 1 (4) |
|  | **Abdominal ultrasounds** |  | |
|  |  | Unremarkable | 4 (15) |
|  |  | Not performed | 22 (81) |
|  |  | Presence of fetuses | 1 (4) |
|  | **Cardiac ultrasounds** |  | |
|  |  | Unremarkable | 1 (4) |
|  |  | Not performed | 26 (96) |
|  | **CT of the entire vertebral  column (n = 3) and head (n = 2)** |  | |
|  |  | Unremarkable | 3 (11) |
|  |  | Not performed | 24 (89) |
|  | **MRI: head (n = 2), cervical (n = 2), thoracolumbar (n = 2)** |  | |
|  |  | Unremarkable | 3 (11) |
|  |  | Not performed | 23 (85) |
|  |  | Multifocal T2 and STIR hyperintense intramuscular lesions in the  affected (rigid) pelvic limbs | 1 (4) |
