# Supplementary material for: Clinical findings and outcome in feline tetanus: a multicentric retrospective study of 27 cases and review of the literature
Source: Front Vet Sci. 2024 Jul 16;11:1425917. doi: 10.3389/fvets.2024.1425917 (PMC11286588; doi:10.3389/fvets.2024.1425917)
Supplement: Supplementary file 9 [file Table_2.DOCX]

Supplementary table 2. Antibiotic treatments and dosage ranges (when available) used to treat tetanus in cats.

| **Antibiotics** | **Dosage range (when available)** | **n = 27 (100%)** |
| --- | --- | --- |
| Metronidazole | 8-15 mg/kg/12h | 20 (74) |
| Amoxicillin/clavulanic acid | 12,5-20 mg/kg/12h | 7 (26) |
| Amoxicillin | 20 mg/kg/12h | 3 (11) |
| Clindamycin | 15-18 mg/kg/12h | 3 (11) |
| Ampicillin | 20 mg/kg/8h | 2 (7) |
| Ampicillin/sulbactam | 20 mg/kg/8h | 1 (4) |
| Penicillin/streptomycin | N.A. | 1 (4) |
| Doxycycline | N.A. | 1 (4) |
