# Supplementary material for: Clinical findings and outcome in feline tetanus: a multicentric retrospective study of 27 cases and review of the literature
Source: Front Vet Sci. 2024 Jul 16;11:1425917. doi: 10.3389/fvets.2024.1425917 (PMC11286588; doi:10.3389/fvets.2024.1425917)
Supplement: Supplementary file 10 [file Table_3.DOCX]

Supplementary table 3. Additional treatments and cares provided to cats affected with tetanus.

| **Myorelaxants** |  | **n = 27 (100 %)** |
| --- | --- | --- |
|  | Diazepam | 6 (22) |
|  | Dantrolene | 3 (11) |
|  | Midazolam | 2 (7) |
|  | Dantrolene + baclofen | 2 (7) |
|  | Magnesium sulfate + diazepam | 1 (4) |
|  | Magnesium sulfate + midazolam | 1 (4) |
|  | Methocarbamol + midazolam | 1 (4) |
|  | Methocarbamol + diazepam | 1 (4) |
|  | Dantrolene + alfuzosine | 1 (4) |
|  | Methocarbamol + diazepam + midazolam | 1 (4) |
|  | Methocarbamol + midazolam  + magnesium sulfate | 1 (4) |
|  | Magnesium sulfate + diazepam + methocarbamol + alfuzosin | 1 (4) |
| **Analgesics** |  | |
|  | Methadone | 3 (11) |
|  | Buprenorphine | 3 (11) |
|  | Transdermal fentanyl | 2 (7) |
|  | Morphine | 1 (4) |
|  | Pregabalin | 1 (4) |
|  | Gabapentine | 1 (4) |
| **Anti-inflammatory drugs** |  | |
|  | Meloxicam | 4 (15) |
|  | Tranexamic acid | 1 (4) |
|  | Metamizole | 1 (4) |
| **Sedative agents** |  | |
|  | Acepromazine | 2 (7) |
|  | Butorphanol | 2 (7) |
|  | Medetomidine | 1 (4) |
|  | Dexmedetomidine | 1 (4) |
|  | Oral cannabidiol | 1 (4) |
| **Nursing measures** |  | |
|  | Daily physiotherapy | 13 (48) |
|  | Acupuncture | 2 (7) |
|  | Maintenance IV fluids | 6 (22) |
|  | Recumbency management | 3 (11) |
|  | Hand feeding | 2 (7) |
|  | Housing of the cat in a dark and quiet rom | 5 (19) |
