# Supplementary material for: Clinical findings and outcome in feline tetanus: a multicentric retrospective study of 27 cases and review of the literature
Source: Front Vet Sci. 2024 Jul 16;11:1425917. doi: 10.3389/fvets.2024.1425917 (PMC11286588; doi:10.3389/fvets.2024.1425917)
Supplement: Supplementary file 11 [file Table_4.DOCX]

| Report | Number of cats described | Breed | Gender | Age (years) | Period of the year | Outdoor access |
| --- | --- | --- | --- | --- | --- | --- |
| *A case of*  *generalized tetanus* (6) | 1 | N.A. | F | 1 | N.A. | N.A. |
| *Changes in  electromyography and F wave responses in two cats with presumed local tetanus* (7) | 2 | DSH  DSH | FN  FN | Young adult  Young adult | N.A.  N.A. | N.A.  N.A. |
| *Localized tetanus*  *in a cat* (10) | 1 | DSH | F | 3 | N.A. | Y |
| *Localized tetanus*  *in two cats after ovariohysterectomy* (11) | 2 | Burmese  DSH | FN  FN | 0,7  0,8 | N.A.  N.A. | N.A.  N.A. |
| *Presumed localized tetanus in two cats* (12) | 2 | DSH  DSH | M  M | 0,8  0,7 | N.A.  N.A. | Y  Y |
| *Tetanus in the cat*  *—an unusual presentation* (13) | 1 | DSH | FN | 1,1 | N.A. | Y |
| *Tetanus in Cat: From Neglected Wound to Neuromuscular Disorder - Case Report* (14) | 1 | DSH | F | 0,5 | N.A. | Y |
| *WHAT IS YOUR DIAGNOSIS? (Localised tetanus in a cat)* (15) | 1 | Persian | MN | 4,5 | N.A. | Y |
| *Tetanus in a cat* (16) | 1 | N.A. | F | 1 | May | Y |
| *Tetanus in a cat* (17) | 1 | DSH | M | 3 | N.A. | N.A. |
| *Tetanus in a cat* (18) | 1 | N.A. | N.A. | N.A. | N.A. | N.A. |
| *Tetanus in a cat* (19) | 1 | DSH | M | 2,5 | January | N.A. |
| *Case report: A*  *severe case of generalized tetanus in a young cat* (20) | 1 | DSH | FN | 0,9 | N.A. | Y |
| *Tetanus in two cats* (21) | 2 | DSH  DLH | FN  F | 8  0,2 | N.A.  N.A. | Y  Y |
| *Tetanus bei einer katze* (22) | 1 | DSH | M | 1 | N.A. | Y |
| *A Case of Tetanus in a Cat* (23) | 1 | DSH | M | 2 | N.A. | Y |
| *Generalized tetanus in a cat* (24) | 1 | DSH | MN | 2 | N.A. | N.A. |
| *Tetanus bei katzen: 3 fallbeschreibungen* (25) | 3 | DSH  DSH  DSH | FN  MN  FN | 3  4  16 | N.A.  N.A.  N.A. | Y  N.A.  N.A. |
| *Three cases of local tetanus* (26) | 2 | DSH  DSH | M  FN | 3  11 | N.A.  N.A. | Y  N.A. |

Supplementary table 4. Epidemiological data from previously reported cases of feline tetanus. *DSH: domestic shorthair cat, DLH: domestic longhair cat, N.A.: not available, F: female, FN: female neutered, M: male, MN: male neutered, Y: yes.*
