# Supplementary material for: Clinical findings and outcome in feline tetanus: a multicentric retrospective study of 27 cases and review of the literature
Source: Front Vet Sci. 2024 Jul 16;11:1425917. doi: 10.3389/fvets.2024.1425917 (PMC11286588; doi:10.3389/fvets.2024.1425917)
Supplement: Supplementary file 12 [file Table_5.DOCX]

| Report | Number of cats described | Presence of a wound | Localization of the wound | Form of tetanus | Outcome |
| --- | --- | --- | --- | --- | --- |
| *A case of*  *generalized tetanus* (6) | 1 | Y | LPL | Generalized (grade II) | Euthanized 9 days after admission |
| *Changes in  electromyography and F wave responses in two cats with presumed local tetanus* (7) | 2 | Y  Y | LPL and LTL  Both TLs | Multifocal (PLs)  Multifocal (TLs, neck) | Normal 1 month after admission  Normal 2 months after admission |
| *Localized tetanus*  *in a cat* (10) | 1 | Y | RTL | Focal (RTL) | Normal 25 days after admission (persistent RTL lameness) |
| *Localized tetanus*  *in two cats after ovariohysterectomy* (11) | 2 | Y  Y | Ovariohysterectomy (left flank incision)  Ovariohysterectomy  (left flank incision) | Multifocal (PLs, LTL, left truncal muscles)  Multifocal (LPL, truncal muscles) | Normal 12 weeks after admission  Improvement of signs for 6 weeks then lost to follow-up |
| *Presumed localized tetanus in two cats* (12) | 2 | Y  N | Right side of the neck | Focal (RTL)  Multifocal (TLs, facial muscles) | Ambulatory on all limbs 1 week after admission (persistent RTL lameness 5 months after admission)  Persistent mild stiffness of TLs 2 months after admission |
| *Tetanus in the cat*  *—an unusual presentation* (13) | 1 | Y | LTL | Multifocal (4Ls) | Normal 3 weeks after admission |
| *Tetanus in Cat: From Neglected Wound to Neuromuscular Disorder - Case Report* (14) | 1 | Y | Left side of the neck | Multifocal (neck, TLs, facial muscles) | Normal 23 days after admission |
| *WHAT IS YOUR DIAGNOSIS? (Localised tetanus in a cat)* (15) | 1 | Y | Ventral surface of the tail, near the anus | Multifocal (PLs, LPL > RPL) | Normal 28 days after admission |
| *Tetanus in a cat* (16) | 1 | Y | History of labor, bite wound on LPL, neutering surgery | Generalized (grade I) | Normal 45 days after admission |
| *Tetanus in a cat* (17) | 1 | Y | LPL | Generalized (grade II) | Cardiac arrest few hours after admission |
| *Tetanus in a cat* (18) | 1 | Y | One of the PLs | Signs spread to all body (no more information available) | The cat recovered (no more information available) |
| *Tetanus in a cat* (19) | 1 | Y | RPL | Generalized (grade II) | Cardiorespiratory arrest 3 days after admission |
| *Case report: A*  *severe case of generalized tetanus in a young cat* (20) | 1 | Y | LPL | Generalized (grade II) | Cardiorespiratory arrest 7 days after admission |
| *Tetanus in two cats* (21) | 2 | Y  Y | RTL  LPL | Generalized (grade I to II)  Generalized (grade II) | Normal 12 weeks after admission  Euthanized 6 days after admission |
| *Tetanus bei einer katze* (22) | 1 | Y | RTL, castration | Generalized (grade II) | Cardiorespiratory arrest 2 days after admission |
| *A Case of Tetanus in a Cat* (23) | 1 | Y | LTL | Generalized (grade I) | Euthanized 2 days after first signs |
| *Generalized tetanus in a cat* (24) | 1 | Y | LPL | Generalized (grade I) | Persistent mild stiff LPL gait 4 weeks after discharge |
| *Tetanus bei katzen: 3 fallbeschreibungen* (25) | 3 | Y  Y  Y | RPL  RTL  Neck | Focal (RPL)  Focal (RTL)  Generalized (grade II) | Normal 56 days after admission  Ambulatory on all limbs 28 days after admission  Ambulatory 27 days after admission |
| *Three cases of local tetanus* (26) | 2 | Y  Y | LTL  LTL | Focal (LTL)  Multifocal (LTL, left side of the neck) | Euthanized 3 days after admission  Normal 84 days after admission |

Supplementary table 5. Clinical data from previously reported cases of feline tetanus (presence and localization of a wound, reported form of tetanus and outcome). *Y: yes, N: no, LPL: left pelvic limb, RPL: right pelvic limb, LTL: left thoracic limb, RTL: right thoracic limb, TLs: thoracic limbs, PLs: pelvic limbs.*
