# Supplementary material for: Clinical findings and outcome in feline tetanus: a multicentric retrospective study of 27 cases and review of the literature
Source: Front Vet Sci. 2024 Jul 16;11:1425917. doi: 10.3389/fvets.2024.1425917 (PMC11286588; doi:10.3389/fvets.2024.1425917)
Supplement: Supplementary file 13 [file Table_6.DOCX]

| Report | Number of cats described | Diagnostic modalities | Pertinent results |
| --- | --- | --- | --- |
| *A case of*  *generalized tetanus* (6) | 1 | Bloodwork | Marked increased in creatinine kinase activity, elevated lactate, mild anemia |
| *Changes in  electromyography and F wave responses in two cats with presumed local tetanus* (7) | 2 | Electrodiagnostic (general anesthesia)  Electrodiagnostic (general anesthesia) | Spontaneous pathological activity in most muscles from the pelvic limbs and tail, occasional MUAPs. Increase in F/M amplitude ratio and F wave duration  MUAPs as single potentials,  doublets, triplets or multiplets recorded simultaneously in antagonist muscles of the neck and front limbs and in tibial cranial muscle in both pelvic limbs. Increase F/M amplitude ratio. |
| *Localized tetanus*  *in a cat* (10) | 1 | Bloodwork  X-ray  Electrodiagnostic (general anesthesia)  Muscle biopsy | Pathological spontaneous activity with sporadic MUAPs in all muscles of the right forelimb. No pathological activity was found in the muscles of the remaining three limbs, the head or the trunk. Nerve conduction velocity of both radial nerves showed normal results. |
| *Localized tetanus*  *in two cats after ovariohysterectomy* (11) | 2 | Bloodwork  x-rays  Toxoplasmosis serology  CSF  EMG (general anesthesia)  Bloodwork  EMG (general anesthesia) | Moderate increase in creatinine kinase activity  Continuous pattern of MUAPs in the pelvic limbs, left thoracic limb and truncal muscles.  Continuous pattern of MUAPs in the left thoracic limb and truncal muscles. |
| *Presumed localized tetanus in two cats* (12) | 2 | Bloodwork  X-rays  CSF analysis  Bloodwork  X-rays  CSF analysis  EMG (general anesthesia) | None  MUAPs that persisted after the insertion of the needle and despite the partial relaxation of the muscles |
| *Tetanus in the cat*  *—an unusual presentation* (13) | 1 | Bloodwork | Marked increased in creatinine kinase activity |
| *Tetanus in Cat: From Neglected Wound to Neuromuscular Disorder - Case Report* (14) | 1 | X-rays | None |
| *WHAT IS YOUR DIAGNOSIS? (Localised tetanus in a cat)* (15) | 1 | Bloodwork  X-rays | None |
| *Tetanus in a cat* (16) | 1 | Bloodwork  Tetanus toxin antibody test via indirect hemagglutination | Detection of antibodies |
| *Tetanus in a cat* (17) | 1 | Not reported |  |
| *Tetanus in a cat* (18) | 1 | Not reported |  |
| *Tetanus in a cat* (19) | 1 | Mouse bioassay | Confirmed presence of the toxin from the wound culture |
| *Case report: A*  *severe case of generalized tetanus in a young cat* (20) | 1 | Bloodwork  X-rays  Culture of the skin lesion | Mild, normocytic, normochromic, non-regenerative anemia  Electrolyte derangements  Increased hepatic parameters  Marked increase in the creatine kinase and Serum Amyloid A  Presence of *Clostridium perfringens* and *Pseudescherichia vulneris* in the wound |
| *Tetanus in two cats* (21) | 2 | Not reported  Not reported |  |
| *Tetanus bei einer katze* (22) | 1 | Mouse bioassay | Confirmed presence of the toxin from the wound culture |
| *A Case of Tetanus in a Cat* (23) | 1 | Culture of the wound | Growth of *Clostridium spp* |
| *Generalized tetanus in a cat* (24) | 1 | Bloodwork | Mild increase in creatine kinase activity |
| *Tetanus bei katzen: 3 fallbeschreibungen* (25) | 3 | Bloodwork  Culture of the wound  Bloodwork  Urine analysis  EMG (general anesthesia)  Bloodwork  Urine analysis  CSF analysis | Growth of *Staphylococcus chromogenes*  and *Clostridium spp*  Continuous MUAPs on the right thoracic limb, more pronounced in the triceps brachii muscle. No anomaly in other muscles.  None |
| *Three cases of local tetanus* (26) | 2 | X-ray  Autopsy  CSF analysis | None  None |

Supplementary table 6. Diagnostic modalities and findings from previous reports of feline tetanus. *EMG: electromyography, CSF: cerebrospinal fluid, MUAPs: motor unit action potentials*
